# Nutritional Composition of Four Edible Grasshopper Species Frequently Consumed in Madagascar: Insights for Nutritional Contribution and Alternative Insect Farming

**DOI:** 10.3390/foods14111848

**Published:** 2025-05-22

**Authors:** Henlay J. O. Magara, Sylvain Hugel, Brian L. Fisher

**Affiliations:** 1 Department of Feed Development, Madagascar Biodiversity Center, Antananarivo 101, Madagascar; hugels@inci-cnrs.unistra.fr; 2 Institut des Neurosciences Cellulaires et Intégratives, Centre National de la Recherche Scientifique, Université de Strasbourg, 67087 Strasbourg, France; 3 Department of Entomology, California Academy of Sciences, San Francisco, CA 94118, USA

**Keywords:** entomophagy, edible grasshoppers, Acriridae, food security, nutrition content

## Abstract

Edible insects are a significant component of traditional diets in Madagascar, where food insecurity and malnutrition persist. This study examines the production parameters and nutritional composition of four laboratory-farmed edible grasshopper species commonly consumed by Malagasy people with the aim of upscaling their farming to mitigate malnutrition. The grasshopper species include: vlei grasshopper (*Paracinema tricolor*), rice grasshopper (*Oxya hyla*), emerald-legged grasshopper *(Eyprepocnemis smaragdipes*), and Madagascan slant-faced grasshopper (*Acrida madecassa*). The study involved the assessment of production parameters (survival rate, developmental time, feed consumed, feed conversion ratio, biomass yield, fecundity, and hatchability). The study also involved analysis of the nutritional content (moisture, protein, fat, ash, fibre, carbohydrates, minerals, amino acids, fatty acids, and vitamins) to evaluate the potential dietary contribution of these grasshoppers. The result show *P. tricolor* had superior survival, faster development, low feed intake, and higher fecundity and hatchability when compared to other species of grasshoppers. *Acrida madecassa* showed the highest biomass yield and feed conversion ratio followed by *P. tricolor*. The results further show that all four species are rich in protein, essential fatty acids, and key minerals, particularly calcium, phosphorus, iron, and zinc. *P. tricolor* exhibited the highest protein and fat content. Moreover, *P. tricolor* showed the highest ash content, suggesting a superior mineral profile. *Acrida madecassa* showed the highest fibre content, reflecting its richness in chitin. These findings provide valuable insights into the nutritional role of grasshoppers in Malagasy diets. Furthermore, they offer reference values for selecting and optimizing the nutrient composition of insect species that are safe and easy to rear, which could serve as a sustainable alternative to wild collection. Future research should explore the bioavailability of nutrients in these species and identify suitable practices to mass rear these species to improve food security in Madagascar.

## 1. Introduction

Ensuring adequate nutrition remains a significant challenge in Madagascar, where current food production systems struggle to meet the dietary needs of 32 million residents [1]. With the population projected to surpass 53 million by 2050, the demand for food will continue to rise, exacerbating existing challenges [2]. Several factors contribute to food insecurity, including limited arable land, poor soil fertility, erratic rainfall, prolonged droughts, cyclones that damage crops and livestock, and high levels of poverty [1,3]. As a result, malnutrition remains a critical public health issue, with 9.4 million children under the age of five experiencing stunting, wasting, or underweight conditions [4,5,6].

To address this growing crisis, it is essential to explore alternative food sources that are nutritionally valuable, climate-resilient, and require minimal land, water, and feed inputs. Among these alternatives, edible insects—particularly Orthopterans—play a significant role in Malagasy diets. Historical evidence suggests that Orthopterans have been consumed in Madagascar for centuries [7,8,9,10,11,12]. Grasshoppers, the most widely consumed Orthoptera in the country [9] and widely consumed across Africa [13,14], are an important seasonal source of nutrition traditionally harvested from the wild. According to a survey by Van Itterbeeck and colleagues, Malagasy people consider 26 species of grasshoppers to be edible [9]. Grasshoppers are traditionally consumed in Madagascar, particularly in specific regions where they are considered a part of the local diet. While these grasshoppers are widely consumed, their commercialization and consumption are not explicitly regulated by food legislation. Data from Orthopterans found in other countries indicate that these insects provide high-quality protein, polyunsaturated fatty acids, and essential micronutrients, including iron, zinc, potassium, and calcium [15,16,17,18,19], thereby contributing significantly to local nutrition.

Most resource-poor Malagasy people preserve their grasshoppers by drying and salting them for future consumption. On the other hand, the most privileged store their grasshoppers in freezers or refrigerators. Surplus grasshoppers are sold alive or in deep-fried form in local markets by men, women, and youths as a source of income. However, the sustainability and safety of relying on wild-harvested grasshoppers as a primary food source remain uncertain. The large-scale collection of wild insects shares similarities with bushmeat consumption, as it exploits natural populations without controlling reproduction, potentially leading to overharvesting and ecosystem imbalances [20,21]. This is particularly likely to occur during times when there is rising demand and increasing market prices, conditions that could drive unsustainable harvesting practices and further threaten insect populations [20,22]. Nevertheless, a primary concern is the availability of grasshoppers, as relying on wild-caught insects for a consistent supply is challenging due to seasonal variations and unpredictable natural population fluctuations. Moreover, the potential bioaccumulation of heavy metals and pesticide residues in wild-caught insects further compounds these challenges, heightening concerns regarding food safety and toxicity [14]. Since the diets of wild insects are uncontrolled, they may bioaccumulate heavy metals and pesticide residues, raising concerns about potential toxicity [23,24]. The mass-rearing of grasshoppers might seem like a viable alternative to wild harvesting; however, this approach remains challenging due to their substantial need for fresh foliage, making them resource-intensive to farm. Additionally, large-scale grasshopper rearing has been associated with significant allergenic risks for workers [25], as well as agricultural concerns, as many grasshopper species, including some classified as locusts, are major crop pests in Madagascar.

The challenges mentioned above may be why there are no grasshopper farms in Madagascar and explain why farmers have preferred to raise cricket and black soldier fly (BSF). However, the advent of successful grasshopper farming in countries such as China and Mexico, where grasshoppers are raised on locally available grasses and agro-byproducts and where their products have a readily available market, has challenged the belief that farming grasshoppers is unsuitable and unsustainable, at least under the specific conditions present in these regions [26,27]. These farms have demonstrated that grasshoppers require considerably fewer resources than traditional livestock, indicating that they might represent a viable option for small-scale farmers—though further evaluation is warranted [26,27]. These grasshoppers are rich in nutrients, which supports their potential as a sustainable source of protein for both human and animal consumption compared to conventional livestock farming [27]. They release less carbon into the environment, lay many eggs, and have a short lifecycle [27]. Additionally, raising grasshoppers in restricted cages or greenhouses can control their spread to stop them becoming pests [26]. Farming these insects also prevents the overharvesting of wild grasshoppers and might even promote their conservation [28]. Moreover, grasshoppers reared on nutritionally well-characterized diets protect them from accumulating heavy metals, pesticides, and plant toxins, making them safe for human consumption [15,29,30]. With the increasing global demand for sustainable protein supply, grasshopper farming might provide a future-proof business model [27]. Despite the challenges, insights from grasshopper farming in China and Mexico justify exploring its potential in Madagascar and Africa, an idea that inspired our study. To evaluate the feasibility of such farming, we first need to assess the production potential and nutritional composition of traditionally consumed Malagasy grasshoppers and their contribution to local diets. Understanding the production potential and nutritional contribution of these grasshopper species will help evaluate their importance to Malagasy nutrition. Additionally, the results can serve as a reference for identifying target nutritional profiles in edible insect species that are safe and easy to rear, potentially offering a sustainable alternative to the collection of wild grasshoppers.

This study, therefore, aims to analyse the macro- and micronutrient content of farmed grasshoppers belonging to four of the more widely consumed species: *Paracinema tricolor* (Thunberg, 1815), *Oxya hyla* (Serville, 1831), *Eyprepocnemis smaragdipes* (Bruner, 1910), and *Acrida madecassa* (Brancsik, 1892) [9].

## 2. Materials and Methods

### 2.1. Grasshoppers

Adult *P. tricolor*, *O. hyla*, *E. smaragdipes*, and *A. madecassa* grasshoppers were obtained from grassland and rice paddies in Madagascar in the ratio of 100 males to 300 females of each species per rearing cage to establish a mother colony. These grasshoppers were selected for rearing since they are a delicacy in Madagascar, have a short lifecycle, are large, feed on locally available grasses and dry agro-byproducts, require little water, do not require a large space for farming, and do not make noise in residential areas. Grasshopper samples were collected in well-ventilated plastic containers (45 cm × 30 cm × 30 cm) (Rectangle Super 2; Aristo Manufacturers Limited, Mumbai, India) while in transit to the Madagascar Biodiversity Center (MBC) (18.9326° S, 47.5254° E; approximately 1280 m above sea level). At MBC, the grasshoppers were transferred into plastic cages (54 cm × 42 cm × 34 cm) (Rectangle Super 2; Aristo Manufacturers Limited, India) that were placed on tables on the balcony on the second floor of the MBC. The inner walls of each container were lined with organza netting supported by a wire frame and sticks for perching to mimic a natural environment, which maximizes the use of each box. The grasshoppers were provided with moist yet firm sandy soil in small plastic containers for depositing their eggs. Eggs laid in pods less than two hours earlier were collected by carefully observing surface signs on the moist sand, such as tiny holes, small cracks, or disturbed patches, and gently digging near them for egg pods using a spoon. After counting, the egg pods were transferred onto moist sand in a plastic container measuring 22 cm × 16 cm × 15 cm (Rectangle Super 2; Aristo Manufacturers Limited, India), with ventilation provided through holes in the lid. The sand was sprayed daily with water from a hand-held pump sprayer to maintain sufficient humidity for successful incubation. The incubation containers were placed on a shelf in the grasshopper-rearing laboratory and monitored every six hours for nymph hatching. The hatched nymphs were fed on vary mena rice (*Oryza sativa* L.) tillers supplemented with kikuyu grass (*Cenchrus clandestinus*) (Hochst. Ex Chiov.) Morrone, grass collected around MBC, and rice bran. The same rice tillers, grass, and rice bran were used throughout the experiment. Before using the grass, measures were taken to avoid contamination by ensuring that the grass was collected from a controlled area and stored properly before use. All samples were visually inspected, and any potentially contaminated material was discarded. Water was given ad libitum in shallow containers with moistened cotton sheets spread out to prevent the insects from drowning. The feed and water were exchanged after two days. The rearing conditions included temperatures that fluctuated between 28 and 32 °C and were controlled by an electric bulb; a 12:12 light/dark photoperiod, and a relative humidity of 65–70%. The grasshoppers were grown for two generations to provide sufficient material for experiments.

### 2.2. Forage Collection

Vary mena rice forage was obtained from an organic farm in Antananarivo. Kikuyu grass was harvested around MBC, where it is grown under controlled conditions. Both forages were harvested during their respective vegetative growth stages to ensure consistency in nutrient content. After collection, the forages were carefully cleaned using tap water to remove any external contaminants. The rice bran was procured from a registered stockiest in Antananarivo, Madagascar.

### 2.3. Experimental Design

The study was conducted under controlled laboratory conditions using a completely randomized design (CRD), with five replicates per species. Each replicate consisted of 400 first-instar nymphs reared in standardized rearing cages (54 cm × 42 cm × 34 cm) that were well ventilated with fine mesh. Thus, a total of 2000 individuals per species (400 individuals × 5 replicates) were used. For the nutritional analyses, insects from all five cages were pooled by species and treatment to create a single composite sample, which was then homogenized and subsampled for laboratory analysis. The experiments were set in a laboratory with the following environmental conditions: 28–32 °C temperature; 65–70% relative humidity; and a 12 h of light:12 h of dark day period. Temperature and relative humidity were recorded using a thermal hygrometer data logger. After each feeding session, cages bearing experimental insects were returned to new positions on the tables to prevent position bias. Parameters measured during this study included the nutritional composition of feeds; production parameters such as the developmental time of *P. tricolor*, *O. hyla*, *E. smaragdipes*, and *A. madecassa* grasshopper juveniles; surviving insects; body mass; body length; quantity of feed consumed; feed conversion ratio; biomass yield; nutrient composition of the different grasshoppers; and fecundity and hatchability. These parameters are explained in the subsections below.

### 2.4. Comparison of Production Parameters

To evaluate the production performance of four grasshopper species under the following controlled laboratory conditions: 28–32 °C temperature; 65–70% relative humidity; and a 12 h of light:12 h of dark day period. Each species was reared in five replicate cages, with each cage containing 400 newly hatched juveniles. All replicates were maintained under identical environmental conditions for 50 days, and grasshoppers were provided with fresh food and water ad libitum throughout the rearing period. The survival rate was calculated by counting the number of individuals that reached adulthood in each replicate and expressing this as a percentage of the initial 400 juveniles. Developmental time was recorded as the number of days from hatching to adult emergence, based on daily observations. Once adults emerged, all surviving individuals were collected and weighed to determine the total fresh biomass per cage, from which the average individual biomass was also calculated. Food consumption was monitored by weighing the food offered and the leftover food daily. The total food consumed per replicate was obtained by subtracting the cumulative weight of uneaten food from the total food offered over the rearing period. The feed conversion ratio (FCR) was then calculated as the total food consumed divided by the total biomass produced, indicating the efficiency of food utilization. For the fecundity assessment, five adult females from each species were randomly selected and paired with males in individual containers. The egg pods laid by each female were collected, counted, and incubated under the same environmental conditions. The number of eggs per female was determined by averaging the number of emerged juveniles from the egg pods of the five females per species. Egg hatchability was determined by recording the number of nymphs hatched from the total eggs laid, expressed as a percentage.

### 2.5. Sample Collection and Preparation for Nutrition Analysis

A total of 500 grasshopper samples per grasshopper species were harvested into separate ziplock bags for nutritional analysis. This encompassed 50 adult males and 50 adult females randomly picked from each cage of the five cages per species, and pooling them, giving a total of 250 males and 250 females for each species of grasshoppers. The harvested grasshoppers were inactivated by snap-freezing before being washed in clean tap water to remove dirt and then freezing them at −20 °C to kill them [28]. Grasshopper samples were removed from the freezer and placed on laboratory benches to thaw before being dried in an oven set at 40 °C for 24 h. Dry samples were blended into a powder using a laboratory blender (Moulinex LM42 *, Argos Manufacturer Limited, Avebury Boulevad, United Kingdom. Afterwards, the milled samples were packed into airtight dark bottles to prevent degradation by light and oxidation. The samples collected for analysis weighed 100 g per species, ensuring that adequate material was available to provide accurate and reproducible results. The grasshopper samples and diet samples were then sent to the International Livestock Research Institute (ILRI) nutrition laboratory in Nairobi, Kenya, for analysis. The grasshopper powders were subjected to various nutrition analyses described below.

### 2.6. Proximate Analysis of Kikuyu Grass, Vary Mena Rice Tillers, Rice Bran, Paracinema tricolor, Oxya hyla, Eyprepocnemis smaragdipes, and Acrida madecassa Grasshoppers

Proximate contents in the kikuyu grass, vary mena rice tillers, rice bran, *P. tricolor*, *O. hyla*, *E. smaragdipes*, and *A. madecassa* grasshopper samples were obtained using the Association of Official Analytical Chemists methods [31]. The percentage moisture in the grasshoppers was determined by drying the samples in an oven set at 135 °C for 2 h (Method No. 930.15) [30]. Ash percentage was determined by burning the samples at 550 °C in a muffle furnace until a constant weight was obtained (Method No. 930.05) [31]. The crude fat content was determined by diethyl ether extraction in a fat extraction unit (SER 148/6; Velp Scientific, Usmate, Italy) following the Randall technique (Method No. 2003.05) [31]. The crude protein content of the powders of the diets and grasshoppers was determined following the Kjeldahl method, and the values were multiplied by a conversion factor of 6.25 for diets and 5.33 for grasshoppers [32]. The crude fibre was evaluated by ignition loss using the remaining weight of the grasshoppers after hydrolysis with acid and alkali solutions (Method number 978.10) [33]. Normal carbohydrate content was determined by subtraction (100% DM—(% moisture + % crude protein + % crude lipid + % crude ash + crude fibre)) [34].

### 2.7. Mineral Composition Analysis of Kikuyu Grass, Vary Mena Rice Tillers, Rice Bran, Paracinema tricolor, Oxya hyla, Eyprepocnemis smaragdipes, and Acrida madecassa Grasshoppers

The kikuyu grass, vary mena rice tillers, rice bran, and *P. tricolor*, *O. hyla*, *E. smaragdipes,* and *A. madecassa* grasshopper powders were ashed and digested in 6 N HCl. After digestion, the content of different minerals such as calcium, magnesium, iron, zinc, phosphorus, potassium, sodium, manganese, and copper was obtained using atomic absorption spectrometry (AAS) (Shimadzu, AA-6300, Tokyo, Japan) following the AOAC procedure [33].

### 2.8. Determination of the Fatty Acid Profile of Paracinema tricolor, Oxya hyla, Eyprepocnemis smaragdipes, and Acrida madecassa Grasshoppers

The fat esterification of *P. tricolor*, *O. hyla*, *E. smaragdipes*, and *A. madecassa* grasshoppers was performed following Soxhlet extraction ISO 12966-2:2011 [35] using a standard 0.25 N methanolic potassium hydroxide. The lipid fraction was concentrated, and fatty acids were transesterified into methyl esters using a standard procedure [35]. The extracted lipids were dissolved in hexane, and a known volume of methanol was added to a base catalyst of sodium methoxide. The mixture was then heated under reflux for a specified duration to ensure complete transesterification. After cooling, the fatty methyl esters were separated by washing with water and concentrated under a stream of nitrogen gas. The resulting fatty acid methyl esters were then subjected to analysis following a modified method of AOAC 996.06 [36] on the Agilent 7890 GC gas chromatograph system (Agilent Technologies, Santa Clara, CA, USA) with a flame ionisation detector temperature set at 250 °C fitted with a Restek column Rt-2560 GC Capillary Column, 100 m, 0.25 mm ID, 0.20 µm from Restek Corporation (Bellefonte, PA, USA). Hexane was applied as the solvent, and a sample volume of 1 µL was injected in the split mode in a ratio of 20:1 to the injector heated at 225 °C. The initial oven temperature was 70 °C (holding time 2 min) before being raised to 225 °C at 5 °C/min (holding time 9 min) and then to 240 °C at 5 °C/min for 15 min. Helium was used as the transporting gas of the fatty esters with a 1.2 mL/min flow rate. The results of fatty acid profiles were identified through the standard Food Industry FAME Mix, cat. # 35077, from Restek Corporation (Bellefonte, PA, USA).

### 2.9. Amino Acid Composition Determination of Paracinema tricolor, Oxya hyla, Eyprepocnemis smaragdipes, and Acrida madecassa Grasshoppers

The type of amino acids in the *P. tricolor*, *O. hyla*, *E. smaragdipes,* and *A. madecassa* grasshoppers was determined following the description of Cheseto and colleagues [37]. In brief, the grasshopper powders (100 mg) were put into 5 mL micro-reaction vials followed by an addition of 2 mL of 6 N HCl and covered after introducing nitrogen gas. The samples were transferred to an oven and hydrolysed for 24 h at 110 °C. After hydrolysis, the mixtures were allowed to evaporate to dryness under a vacuum. The dry hydrolysates were reconstituted in 1 mL 0.01% formic acid/acetonitrile (95:5), vortexed for 30 s, sonicated for 30 min, and then centrifuged at 14,000 rpm to form supernatants. Then supernatants were analysed by LC-MS. The same procedure was performed to determine basic amino acids by substituting 6 N NaOH for 6 N HCl.

The reconstituted supernatants were injected into an Agilent system 1100 series (St. Louis, MO, USA) using a ZORBAX SB-C18, 4.6 × 250 mm, 3.5 µm column, operated at 40 °C for chromatography separation. The mobile medium was water (A) and 0.01% formic acid in acetonitrile (B). The following gradient was used: 0–8 min, 10% B; 8–14 min, 10–100% B; 14–19 min, 100% B; 19–21 min, 10–100% B; 21–25 min, 10% B. The flow rate was kept constant at 0.5 mL min^−1^, and the injection volume was 3 µL. The LC was interfaced with a quadrupole mass spectrometer. The mass spectrometer was operated in ESI-positive mode at a mass range of m/z 50–600 at 70 eV cone voltage.

Serial dilutions of the authentic standard containing 18 amino acids (1–105 µg/µL, Sigma–Aldrich, St. Louis, MO, USA) were similarly analysed by LC-MS to generate the linear calibration curves (peak area vs. concentration) used for external quantification. Amino acid analysis was repeated three times using different batches of samples.

### 2.10. Determination of the Vitamins of Paracinema tricolor, Oxya hyla, Eyprepocnemis smaragdipes, and Acrida madecassa Grasshoppers

The quantities of Vitamins A, D, and E (fat-soluble) and B1, B2, B3, B5, B6, B9, and B12 (water-soluble) in the grasshoppers were determined. The study followed the protocol for fat-soluble vitamin analyses [38]. During the analysis, 300 mg of each grasshopper sample was introduced into a 10 mL glass bottle containing hexane, methanol, and distilled deionized water at a ratio of 2:1:2.5 mL. The mixture was vortexed for 30 s before being sonicated for 30 s in a water bath set at 70 °C. After sonication, the mixture was centrifuged at 1400 revolutions per minute for five minutes to form a supernatant. The obtained supernatant was then passed through sodium methoxide to dry before being evaporated to dryness under a gentle flow of nitrogen gas. Afterwards, the dry supernatant was derivatized to remove any remains of fatty acid methyl esters to minimize the matrix interference. The fat-soluble vitamins in the supernatant were analysed by injecting 1.0 µL GC-MS on a 7890A gas chromatograph connected to a 5975C mass selective detector (Agilent Technologies Inc., Santa Clara, CA, USA). The GC was fitted with a (5%-phenyl)-methylpolysiloxane (HP5 MS) low-bleed capillary column (30 m × 0.25 mm i.d., 0.25 μm; J and W, Folsom, CA, USA). Helium was used as the transporting gas at a flow rate of 1.25 mL per minute. The temperature in the oven ranged between 35 °C and 285 °C, with the start temperature maintained for 5 min and a rise at 10 °C per minute to 280 °C, which was maintained for 20 min and 40 s. The mass selective detector was kept at an ion supply temperature of 230 °C and a quadrupole temperature of 180 °C. Electron impact (EI) mass spectra were attained at an acceleration energy of 70 eV. Fragment ions were analysed between a 40 and 550 m/z mass range in full scan mode. The filament and delay time was programmed at 3.3 min. Serial dilutions of the authentic standard α-tocopherol (≥95.5% purity) (0.1–100 ng/μL, Sigma-Aldrich, St. Louis, MO, USA) were analysed by GC-MS in full scan mode to produce linear calibration curves (peak area vs. concentration), which resulted in a coefficient of determination of R = 0.9999. The regression equation was used for the external quantification of the different selected fat-soluble vitamins (Retinol, γ-tocopherol, α-tocopherol, and provitamin D).

These compounds were identified by comparing mass spectral data and retention times with those of authentic standards and reference spectra published by mass spectral (MS) library databases: National Institute of Standards and Technology (NIST) 05, 08, and 11. The samples were analysed in triplicate, with each replicate collected from a different batch of respective samples.

Determination of water-soluble vitamins was carried out according to the previously described method [38]. Briefly, 100 mg of each cricket sample was transferred into a 50 mL Falcon tube containing 25 mL distilled deionized water (25 mL), vortexed for 20 s, sonicated for 15 min, and the mixture filtered through 0.2 μm filters into Ultra Performance Liquid Chromatography (UPLC) vials and analysed by Shimadzu UPLC-DAD. The chromatographic analysis was performed on a LC-30AC with Nexera column oven CTO-30A, (Shimadzu, Tokyo, Japan) fitted with a Phenomenex C18 Column Synergi 100 mm × 3.00 mm, 2.6 μm polar (Phenomenex, Torrance, CA, USA) at 30 °C. The mobile phase consisted of two phases, A: 25 mM phosphate buffer; B: 7:3 *v*/*v* acetonitrile-mobile phase A. The total run time was 12 min with a flow rate of 0.4 mL/min. Stock solutions of 1.0 mg/mL were prepared by dissolving the individual water-soluble vitamin standards in distilled water except for Vitamin B2 (dissolved in 5 mM potassium hydroxide) and Vitamin B9 (dissolved in 20 mM potassium hydrogen carbonate). Serial dilutions of the stock solution (2–15 μg/mL) for the 5 water-soluble vitamins were also analysed by UPLC-DAD, giving an R^2^ of 0.996 or greater. These regression equations were used for the external quantification of the different water-soluble vitamins. All determinations were carried out in triplicate from different batches of the respective samples.

### 2.11. Statistical Analysis

Before the data were analysed, normality and homogeneity of variance tests was applied to the data using the Shapiro–Wilk (*p* > 0.05) test for normality and Levene’s test (*p* > 0.05) for the homogeneity of variances. The assumptions were largely satisfied for the tested variables. A one-way analysis of variance (ANOVA) was applied for survival rate, development time, fecundity, hatchability, feed consumed, feed conversion ratio, and biomass yield and proximate composition, mineral elements, amino acids, fatty acids, and vitamin data, followed by the Student–Newman–Keuls test for means comparison. The data were analysed using R version 4.4.2 (Core Team) [39] statistical software. The threshold of statistical significance was set at *p* < 0.05.

## 3. Results

### 3.1. Proximate Composition and Mineral Element Concentrations of Kikuyu Grass Forage, Vary Mena Rice Forage, and Rice Bran.

The proximate and mineral compositions of kikuyu grass forage (*Cenchrus clandestinus*), vary mena rice forage (*Oryza sativa*), and rice bran were compared (Table 1). Substantial differences in nutrient content were observed among the three feed sources. Moisture content was significantly higher in kikuyu grass (77.80%) and vary mena rice forage (76.20%) compared to rice bran (11.40%). Crude protein levels were highest in kikuyu grass (200.0 g/kg DM), followed by vary mena (180.0 g/kg DM), and lowest in rice bran (130.0 g/kg DM). Conversely, rice bran exhibited the highest fat content (155.0 g/kg DM), while both kikuyu and vary mena contained substantially lower amounts (20.0 and 18.0 g/kg DM, respectively). Crude fibre was greatest in kikuyu grass (280.0 g/kg DM), followed by vary mena (250.0 g/kg DM), and lowest in rice bran (77.0 g/kg DM). Carbohydrate content was highest in rice bran (554.6 g/kg DM), moderate in vary mena (390.8 g/kg DM), and lowest in kikuyu grass (332.2 g/kg DM). The total ash content ranged from 72.0 g/kg DM in rice bran to 90.0 g/kg DM in kikuyu grass.

Regarding macro-mineral composition, calcium was most abundant in kikuyu grass (4.2 g/kg DM), while phosphorus and magnesium were highest in rice bran (12.5 and 5.7 g/kg DM, respectively). Potassium levels were higher in kikuyu (18.0 g/kg DM) and vary mena (17.2 g/kg DM) than in rice bran (8.7 g/kg DM). Sodium was relatively low in all samples, ranging from 0.3 g/kg DM in rice bran to 0.8 g/kg DM in kikuyu grass. For trace minerals, iron content was highest in kikuyu grass (120.0 mg/kg DM), followed closely by vary mena (115.0 mg/kg DM), and was lowest in rice bran (108.0 mg/kg DM). Rice bran contained the highest concentrations of copper (6.3 mg/kg DM) and zinc (33.5 mg/kg DM), while Kikuyu grass had the highest manganese content (70.0 mg/kg DM).

These findings highlight the complementary nutritional roles of these feed materials to the grasshoppers, with kikuyu grass and vary mena rice forage serving as protein- and fibre-rich roughages and rice bran contributing high energy and mineral density. These differences suggest that the selection between the two forages and rice bran could be optimized based on the specific nutritional requirements of the target species.

### 3.2. Comparison of Production Parameters of Paracinema tricolor, Oxya hyla, Eyprepocnemis smaragdipes, and Acrida madecassa Edible Grasshoppers

The results for production parameters of the four edible grasshopper species in Madagascar are presented in Table 2. Survival to adult stage differed significantly among the species. *Paracinema tricolor* demonstrated the highest mean survival rate (90.0%), while *E. smaragdipes* exhibited the lowest (77.7%) (Table 2). Development time also varied significantly among the grasshopper species (Table 2). *P. tricolor* and *O. hyla* reached adulthood faster, taking 41.1 and 42.0 days, respectively. In contrast, *E. smaragdipes* and *A. madecassa* took longer periods to develop, 46.2 and 48.4 days, respectively. Total feed consumption and the feed conversion ratio (FCR) showed significant variation among the grasshoppers (Table 2). *Acrida madecassa* proved the most efficient feed converter with an FCR of 1.44, followed by *P. tricolor* (1.84), *E. smaragdipes* (2.23), and *Oxya hyla* (2.58). Biomass yield was significantly influenced by the species of grasshopper (Table 2). The highest biomass yield was recorded in *A. madecassa* (846.3 g per replicate), followed closely by *P. tricolor* (574.0 g), with *E. smaragdipes* (515.9 g) and *Oxya hyla* (403.4 g) yielding comparatively less. Fecundity, measured as the mean number of eggs laid per female, also varied significantly across the four grasshopper species (Table 2). *Paracinema tricolor* ranked highest, with 276.4 eggs per female, followed by *O. hyla* (260.4 eggs), *A. madecassa* (240.0 eggs), and *E. smaragdipes* (218.6 eggs) (Table 2). Similarly, egg hatchability was significantly different across species (Table 2). The highest hatchability rate was observed in *P. tricolor* (92.2%), with *Oxya hyla* (90.1%), *A. madecassa* (88.1%), and *E. smaragdipes* (86.0%) following in descending order.

### 3.3. Proximate Composition of Paracinema tricolor, Oxya hyla, Eyprepocnemis smaragdipes, and Acrida madecassa Grasshoppers

**Figure 1:**
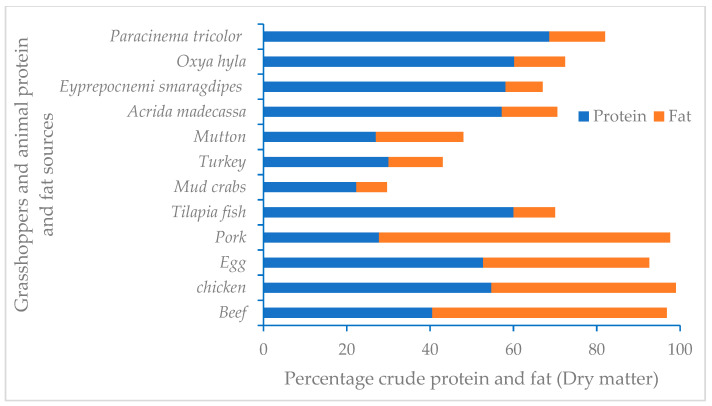
Comparison of the crude protein and fat values of grasshoppers *Paracinema tricolor*, *Oxya hyla*, *Eyprepocnemis smaragdipes*, and *Acrida madecassa* and selected conventional protein sources eaten in Madagascar. The protein and fat data for the conventional sources were obtained from the USDA (United States Department of Agriculture) [40].

The proximate and gross energy content (on a dry matter basis (DM)) of *P. tricolor*, *O. hilya*, *E. smaragdipes*, and *A. madecassa* are presented in Table 3. The proximate composition and energy values varied significantly among the grasshoppers studied. The moisture content of the grasshoppers varied between 2.0% and 9.0%. *Paracinema tricolor* had the least moisture and fibre content of the grasshoppers but contained the highest protein (68.6%), fat (13.8%), fibre and ash (9.7%) content compared with other grasshoppers. *Oxya hyla* had the highest carbohydrate content, while *P. tricolor* had the lowest. *Oxya hyla* and *P. tricolor* contained the most energy compared to other grasshoppers. *Acrida madecassa* had the highest fibre content. The crude protein and fat values of the grasshopper species studied were comparable to those of livestock products consumed by Malagasy people (Figure 1).

### 3.4. Mineral Elements in Paracinema tricolor, Oxya hyla, Eyprepocnemis smaragdipes, and Acrida madecassa Grasshoppers

The mineral values of the *P. tricolor*, *O. hyla*, *E. smaragdipes*, and *A. madecassa* grasshoppers per 100 g dry matter are shown in Table 4. The mineral elements differed significantly across the grasshopper species. Potassium dominated the mineral content of the four grasshoppers (48.1–64.2% of all minerals). Excessive potassium levels can potentially lead to imbalances, especially if other essential minerals are present in insufficient quantities. However, the potassium levels observed in this study fall within the typical range for many insect species. The rest of the mineral elements ranged as follows: phosphorus (16.8–29.7%), calcium (2.0–12.7%), sodium (2.2–9.2%), magnesium (4.3–5.8%), zinc (1.0–1.2%), iron (0.4–0.7%), manganese (0.1–0.4%), and copper (0.1–0.3%). *Paracinema tricolor* had higher magnesium, iron, calcium, phosphorus, zinc, potassium, manganese, and sodium content than the other grasshopper species. The highest amount of copper was recorded in the *Oxya hyla* grasshopper. Maintaining a balanced ratio between phosphorus and calcium minerals is important, as an excess of phosphorus relative to calcium can interfere with calcium absorption and lead to imbalances. Although the phosphorus-to-calcium ratio in the grasshoppers varied, this could potentially pose a concern, particularly in the context of formulating insect-based diets for human consumption. Future studies should explore the optimal phosphorus-to-calcium ratio in insect diets to ensure nutritional balance.

### 3.5. Fatty Acids in Paracinema tricolor, Oxya hyla, Eyprepocnemis smaragdipes, and Acrida madecassa Grasshoppers

Fatty acids varied significantly across the four species of grasshopper. The fatty acids in *P*. *tricolor*, *O*. *hyla*, *E*. *smaragdipes*, and *A*. *madecassa* grasshoppers fed per 100 g dry mass are shown in Table 5. The *Paracinema tricolor*, *O*. *hyla*, *E*. *smaragdipes*, and *A*. *madecassa* grasshoppers contained predominantly linoleic acid (38.92–43.7% of total fats), oleic acid (27.2–35.7%), palmitic acid (10.6–17.3%), stearic acid (8.1–11.3%), myristic acid (0.4–3.3%), arachidic acid (1.3–2.3%), behenic acid (1.1–1.4%), margaric acid (0.4–1.4%), lignoceric acid (0.02–1.4%), alpha-linolenic acid (0.3–0.5%), and lauric acid (0.1–0.4). *Paracinema tricolor* exhibited the highest linoleic acid, alpha-linolenic acid, and margaric acid contents compared with the other grasshoppers. *Oxya hyla* had more stearic acid, myristic acid, and lauric acid than the other grasshoppers. *Eyprepocnemis smaragdipes* contained the highest oleic acid levels compared with the rest of the grasshoppers. The highest palmitic acid, behenic acid, and arachidic acid contents were observed in *A. madecassa*. *Oxya hyla* had the lowest ratio of polyunsaturated fatty acid (PUFA) to saturated fatty acid (SFA), while the highest was observed in *P. tricolor.* All in all, the grasshoppers had PUFA-to-SFA ratios recommended for good human health. The highest essential fatty acid level was noted in *P. tricolor*, and the lowest was recorded in *E. smaragdipes.*

### 3.6. Amino Acids in Paracinema tricolor, Oxya hyla, Eyprepocnemis smaragdipes, and Acrida madecassa Grasshoppers

The amino acids in *P. tricolor*, *O. hyla*, *E. smaragdipes*, and *A. madecassa* grasshoppers fed per 100 g dry mass are shown in Table 6. The amino acids and nonessential amino acids differed significantly in the four species of grasshoppers (Table 6). All four grasshoppers contained seven essential amino acids, as shown in Table 6. The values of the seven essential amino acid varied significantly among *P. tricolor*, *O. hyla*, *E. smaragdipes*, and *A. madecassa*. The nonessential amino acids recorded in *P. tricolor*, *O. hyla*, *E. smaragdipes*, and *A. madecassa* are presented in Table 6. Leucine was the predominant amino acid in *P. tricolor*, *O. hyla*, *E. smaragdipes*, and *A. madecassa*. The highest amounts of histidine, isoleucine, lysine, methionine, phenylalanine, leucine, arginine, glutamine, glutamic acid, tyrosine, and hydroxyproline were also recorded in *P. tricolor* (Table 6). Valine and proline levels were higher in *O. hyla* than in the other grasshoppers (Table 6).

### 3.7. Vitamins in Paracinema tricolor, Oxya hyla, Eyprepocnemis smaragdipes, and Acrida madecassa Grasshoppers

The vitamins in *P. tricolor*, *O. hyla*, *E. smaragdipes*, and *A. madecassa* grasshoppers per 100 g dry mass are shown in Table 7. The vitamin content varied significantly in the four species of grasshoppers (Table 7). The grasshoppers studied exhibited seven water- and fat-soluble vitamins, as shown in Table 7. *Paracinema tricolor* was richest in vitamins A, E, C, B1, B3, and B9, while *A. madecassa* contained the smallest quantity of vitamins. *Oxya hyla* had the most vitamin B2 of the grasshoppers. Vitamin B3 was the predominant vitamin in *P. tricolor*, *O. hyla*, *E. smaragdipes*, and *A. madecassa* (Table 7).

## 4. Discussion

This study provides the first detailed comparison of the production parameters and nutritional assessment of *P. tricolor*, *O. hyla*, *E. smaragdipes*, and *A. madecassa*, four grasshopper species frequently consumed by Malagasy people. The results highlight their substantial variation in production parameters, protein content, valuable lipid profile, and significant mineral and vitamin contributions, demonstrating these species could become an important source of nutrition in local diets.

The present study demonstrated significant differences in production performance among the four grasshopper species reared under identical conditions. *Paracinema* consistently outperformed the other species of grasshoppers, showing superior survival, faster development, greater biomass yield, higher fecundity, and more efficient feed conversion. These outcomes demonstrate that the species has good biological potential for insect farming. The efficient performance of *P. tricolor* could be attributed to its ecological adaptability and robust physiological traits. Likewise, *O. hyla* also showed strong production parameters, making it a secondary candidate for mass culture systems. In contrast, *E. smaragdipes*, and *A. madecassa* lagged behind in multiple metrics, possibly due to species-specific nutritional demands or sensitivity to laboratory conditions. The high fecundity and hatchability observed in *P. tricolor* are especially important for scaling operations, ensuring consistent stock renewal and productivity. The low FCR of *P. tricolor* further enhances its economic viability, offering a better return on feed input, a major cost factor in insect farming. These findings support previous reports suggesting that orthopteran species vary widely in captive adaptability and productivity and reinforce the need for strategic species selection in commercial entomoculture systems (8).

The protein content was high across all species, with *P. tricolor* exhibiting the highest levels. This suggests that grasshoppers can be a meaningful source of dietary protein, especially in regions where protein–energy malnutrition is a concern. The protein content range recorded in the four grasshopper species was within the 43–77% reported for other grasshoppers [16,41]. Further, the protein quality of grasshoppers in this study was comparable to that of conventional protein sources such as ground beef, chicken, goat meat, mutton, crabs, fish, and eggs on a dry matter basis [8,17]. Therefore, the protein content in the four grasshoppers is sufficient to substitute for conventional protein sources because these species constitute a high-protein food source that reduces hunger [42]; helps consumers gain muscle mass and strength [43]; provides better bone health and reduces the risks of osteoporosis and fractures with aging; reduces cravings and the desire for late-night snacking [44,45]; could lower blood pressure [46]; helps with weight loss and long-term healthy weight maintenance [47]; improves tissue repair after injury; and helps older people maintain their physical fitness [48]. The crude protein composition in our study demonstrates that about 36.0 g of *P. tricolor*, 39.9 g of *O. hyla*, 41.3 g of *E. smaragdipes*, and 42.0 g of *A. madecassa* grasshoppers would be sufficient to supply the RDI for children. At the same time, 67.1 g and 81.7 g of *P. tricolor*, 76.4 g and 93.0 g of *O. hyla*, 79.2 g and 96.4 g of *E. smaragdipes*, and 80.5 g and 98.0 g of *A. madecassa* grasshoppers would be adequate to meet the RDI of protein for adult males and females, respectively, which ranges between 46 and 56% [49]. The presence of essential amino acids, particularly lysine, is noteworthy given the predominance of rice-based diets in Madagascar, which are often deficient in this amino acid [50]. Compared to amino acids from animal meats, the four grasshoppers have more of the essential amino acid valine than pork and broiler chicken but are similar to these foods in terms of all other amino acids. Most people consume plain rice with water (rony), while the fortunate accompany their rice with a relish of vegetables. Those whose diets consist primarily of these staples are at risk of protein–energy malnutrition (PEM); those with serious PEM are highly susceptible to diseases such as tuberculosis and gastroenteritis [51]. Serious cases of PEM, including kwashiorkor, can cause skeletal muscle loss and be fatal. Undernutrition is reported to cause more than 45% of the mortality (more than 3 million deaths) among children under five years in developing countries [52]. Malnutrition remains a major problem in Madagascar today, where chronic undernutrition causes distress to about 48% of the population [1,6] and contributes to 6.6% of deaths in children under 5 years old [53]. Therefore, for better access to essential amino acids, including lysine, there is a need to supplement the rice-heavy Malagasy diet with other foods [54]. A diet featuring grasshoppers, which are rich in essential amino acids, can ameliorate these nutritional deficiencies.

Fat content varied among the grasshopper species, with *P. tricolor* containing the highest fat levels. Fats are important not only as an energy source but also as a provider of essential fatty acids and some types of fat-soluble vitamins required by human beings [16]. The fat content range of the grasshoppers in this study is consistent with that reported for other grasshoppers (4.20–22.20%) [16]. Moreover, the fat content of the grasshoppers in the current study was comparable to that in fish, turkey, and mud crabs but lower than that in the beef, chicken, mutton, eggs, and pork commonly consumed by Malagasy people. This low fat content makes grasshoppers a superfood, since they are low in cholesterol compared to livestock fats. The presence of essential fatty acids, including omega-3 and omega-6, further enhances the nutritional value of these grasshoppers, as these compounds are essential for cardiovascular and cognitive health [8]. Linoleic acid constituted the dominant fatty acid, followed by oleic and palmitic acid in all four grasshopper species studied. This result is similar to the findings by Paul and colleagues [16], who reported linoleic acid as the major PUFA fatty acid, followed by oleic acid (MUFA) and palmitic acid (SFA), in other grasshopper species. The ratio of PUFA to SFA varied according to the grasshopper species. In this study, the PUFA-to-SFA ratio was low and within the recommended of 5:1 ratio for good human health [19]. This underscores the significant contribution of edible insects as foods with the potential to facilitate human health by producing cardiovascular-friendly oils, hence reducing high blood pressure rates. Similarly, low PUFA-to-SFA ratios help prevent other diseases such as obesity, hypertension, asthma, diabetes mellitus, and some cancers [55]. These fatty acids are recommended for cooking since they serve as nutrient sources. Moreover, they undergo derivatization into hydroperoxides during cooking, leading to the formation of aroma compounds that influence sensory acceptance (van Huis, A. (2013)) [56]. The essential fatty acid contents varied across the grasshoppers in this study. The level of essential fatty acids (EFAs) in our study was comparable to the results reported for edible grasshoppers and crickets [16,28]. Essential fatty acids in edible grasshoppers are critical as they demonstrate the quality of insect fats [22]. The n-6 and n-3 fatty acid ratio is mostly 5.8:10 to 57.7:10 [57]. Maintaining an omega-6-to-omega-3 (ω6/ω3) fatty acid ratio closer to 4:1 or lower is considered beneficial for human health, as it helps reduce the risk of chronic inflammatory diseases such as cardiovascular disease, obesity, and certain cancers. A balanced ratio supports anti-inflammatory processes, promotes cardiovascular and brain health, and aligns with the evolutionary human diet, which traditionally featured lower ω6/ω3 ratios. Modern diets, often rich in omega-6 and deficient in omega-3, tend to exhibit imbalanced ratios exceeding 10:1, contributing to a higher incidence of inflammation-related conditions [58]. The wide variation in PUFAs and SFAs and in n-6 and n-3 fatty acids in all the grasshopper species could be a result of incorporating rice bran as feed for the grasshoppers. Dry diets have been reported to cause a great variation fatty acids, and this highlights the necessity of proper selection of the feed for farmed grasshoppers for good human health [59].

Ash analysis revealed that *P. tricolor* had the highest ash content, suggesting a superior mineral profile. Iron, calcium, phosphorus, and zinc were particularly abundant across all species. Given that deficiencies in iron, magnesium, calcium, and zinc are prevalent in Madagascar, regular consumption of these grasshoppers may contribute to improved mineral intake, especially for populations with limited access to diverse food sources. The mineral content of the grasshoppers in our study exceeded that found in various delicacies of the Malagasy people, including fish, turkey, mud, beef, chicken, mutton, eggs, and pork. Consuming whole grasshoppers, grasshopper powder, or grasshopper-based meals significantly contributes to the recommended dietary intake (RDI) of many essential minerals. For instance, the grasshopper powder may provide 10–10.5%, 47.8–111.9%, 11.4–14.9%, and 66.5–73.7% of the RDIs for iron, zinc, calcium, and phosphorus, respectively, as determined by FAO/WHO [60]. Given that a lack of minerals, such as calcium, iron, and zinc, remains a critical concern in Madagascar [61], leading to widespread deficiency diseases like rickets and anaemia [62], consuming *P. tricolor*, *O. hilya*, *E. smaragdipes*, and *A. madecassa* grasshoppers early in life may provide a remedy. Grasshopper powders or food blends made from grasshopper powders can help mitigate micronutrient deficiencies among school-going children and pregnant women.

In the current study, the carbohydrate content, which is known to be an energy source, was relatively low, consistent with previous studies on edible insects. Studies conducted on edible insects in Thailand showed low carbohydrate levels ranging from 7 to 16%, similar to some insects in Nigeria that had a carbohydrate content of 7–20% [63]. Chitin, a polysaccharide found in insect exoskeletons, may provide additional health benefits, such as immune modulation, gut health support, heart health, and blood sugar regulation [64]. Moreover, because insect carbohydrates are classified as low carbohydrate–high protein (LC-HP), consuming edible grasshoppers should help reduce the risks of cardiovascular disease and body weight maintenance [49,63].

Vitamin analysis confirmed that these grasshoppers are rich in key vitamins, including A, B-complex, C, and E, further supporting their potential contribution to micronutrient intake. Vitamin A levels are particularly relevant in Madagascar, where deficiency-related diseases such as night blindness remain a concern [8]. Our study reveals that the vitamin A levels in the four studied grasshopper species were higher than that of the green tree locust *Cytacanthacris aeruginosus* (1 mcg/100 g) [65], the painted grasshopper *Zonocerus variegatus* (1st instar larvae) (111.79 mcg/100 g) [63], and short horned grasshoppers (6.82 mcg/100 g). However, the vitamin A levels obtained in this study were lower than for *Z. variegatus* (adult) (814.4) [66]. Therefore, to achieve the recommended daily intake, children would need to consume 149.62 mcg and adults 179.53 mcg of *P*. *tricolour*, 170.40 mcg–204 mcg of *O. hyla*, 176.62–211.95 mcg of *E*. *smaragdipes*, and 179.40–215.29 mcg of *A. madecassa*. Children in Madagascar and other resource-poor countries are at dire risk of vitamin A deficiency (VAD). This qualifies *P*. *tricolour*, *O*. *hyla*, *E*. *smaragdipes*, and *A. madecassa* as promising and sustainable sources of this vitamin [19].

## 5. Conclusions

While these results confirm the potential of the production and nutritional importance of farmed grasshoppers in Malagasy diets, large-scale reliance on their collection from the wild is problematic. Overharvesting might disrupt local ecosystems, while pronounced seasonality, natural population fluctuations, and the potential bioaccumulation of heavy metals and pesticide residues further compromise a consistent, safe supply. Instead, these findings can guide the selection of edible insect species that are safe and easy to farm. By identifying species with nutritional profiles similar or superior to conventional foods that can be farmed sustainably at the household or community levels, it may be possible to provide a more stable and ecologically responsible source of edible insects for Malagasy populations. The findings of this study, therefore, reveal that *P. tricolor* exhibits the most favourable characteristics for mass rearing for nutrient production, followed closely by *Oxya hyla*. Both species demonstrate potential for sustainable insect farming, with implications for food security, feed industry applications, and circular agriculture. The selection of a high-performing species is essential to optimize yield, efficiency, nutrition, and profitability in grasshopper farming systems. Further research should focus on the bioavailability of nutrients in both wild-collected and rearable insect species, as well as strategies to integrate insect farming into existing food systems.

## Figures and Tables

**Table 1:** Proximate composition and mineral composition (in dry matter basis) of kikuyu grass, vary mena rice forage, and rice bran used for rearing *Paracinema tricolor*, *Oxya hyla*, *Eyprepocnemis smaragdipes*, and *Acrida madecassa*.

Nutrient Components and Unts	Kikuyu Grass Forage	Vary Mena Rice Forage	Rice Bran
Proximate			
Moisture (%)	77.80 ± 0.92	76.20 ± 0.75	11.40 ± 0.39
Protein (g/kg DM)	200.00 ± 0.45	180.00 ± 3.50	130.00 ± 2.15
Fats (g/kg DM)	20.00 ± 1.22	18.00 ± 1.01	155.00 ± 2.05
Ash (g/kg DM)	90.00 ± 2.5	85.00 ± 2.20	72.00 ± 1.84
Crude fibre (g/kg DM)	280.00 ± 7.10	250.00 ± 6.20	77.00 ± 1.98
Carbohydrate (g/kg DM)	332.20 ± 9.02	390.80 ± 8.50	554.60 ± 3.35
Mineral elements			
Calcium (g/kg DM)	4.20 ± 0.20	3.80 ± 0.20	1.32 ± 0.02
Phosphorus (g/kg DM)	2.10 ± 0.10	2.01 ± 0.08	12.48 ± 0.45
Magnesium (g/kg DM)	1.01 ± 0.05	0.92 ± 0.04	5.85 ± 0.30
Potassium (g/kg DM)	18.03 ± 0.71	17.22 ± 0.50	8.68 ± 0.32
Sodium (g/kg DM)	0.80 ± 0.04	0.72 ± 0.03	0.28 ± 0.01
Iron (mg/kg DM)	120.00 ± 6.02	115.00 ± 4.58	108.00 ± 5.25
Copper (mg/kg DM)	5.50 ± 0.40	5.02 ± 0.30	6.25 ± 0.20
Zinc (mg/kg DM)	22.00 ± 0.72	20.04 ± 0.60	33.54 ± 0.58
Manganese (mg/kg DM)	70.00 ± 3.00	65.00 ± 3.10	45.50 ± 0.40

Results are expressed as mean values ± standard deviation of three determinations on a dry matter (DM) basis, except moisture, which is expressed in fresh weight.

**Table 2:** Production parameters of *Paracinema tricolor, Oxya hyla*, *Eyprepocnemis smaragdipes*, and *Acrida madecassa* edible grasshoppers.

Grasshopper Species	Survival Rate to Adult (%)	Development Time to Adult (Days)	Feed Consumed (g)	Feed Conversion Ratio	Biomass Yield (g)	Fecundity (Eggs per Female)	Hatchability (%)
*Paracinema tricolor*	90.04 ± 0.18 a	41.14 ± 0.32 a	1050.40 ± 0.54 a	1.84 ± 0.01 b	573.99 ± 1.13 b	276.42 ± 0.51 a	92.22 ± 0.17 a
*Oxya hyla*	85.12 ± 0.36 b	42.02 ± 0.29 b	1071.01 ± 5.34 b	2.58 ± 0.11 d	403.56 ± 4.28 d	260.39 ± 1.21 b	90.10 ± 0.22 b
*Eyprepocnemis* *smaragdipes*	77.72 ± 0.32 d	46.20 ± 0.34 c	1151.00 ± 4.30 c	2.23 ± 0.01 c	515.93 ± 2.06 c	218.64 ± 0.51 d	85.98 ± 0.17 d
*Acrida madecassa*	80.76 ± 0.53 c	48.42 ± 0.25 d	1217.20 ± 6.20	1.44 ± 0.01 a	846.26 ± 5.50 a	240.03 ± 0.71 c	88.06 ± 0.20 c
F	212.90	132.90	257.7	83.23	371.60	1014.00	199.70
*p*	<0.0001	<0.0001	<0.0001	<0.0001	<0.0001	<0.0001	<0.0001
df	3, 16	3, 16	3, 16	3, 16	3, 16	3, 16	3, 16

Results are expressed as mean values ± standard error of five replicates. Means with different letters in each column significantly differ at *p* < 0.05.

**Table 3:** Proximate composition (% dry matter basis) of *Paracinema tricolor*, *Oxya hyla*, *Eyprepocnemis smaragdipes*, and *Acrida madecassa* edible grasshoppers and recommended daily intakes.

Grasshopper	Nutrient						
	Moisture (%)	Protein (%)	Lipids (%)	Fibre (%)	Ash (%)	Carbohydrate (%)	Energy (Kcal/kg)
*Paracinema* *tricolor*	1.95 ± 0.16 a	68.58 ± 0.49 a	13.38 ± 0.74 a	3.89 ± 0.07 c	9.76 ± 0.07 a	2.44 ± 0.02 c	4044.90 ± 45.56 a
*Oxya hyla*	2.54 ± 0.23 b	60.22 ± 0.94 b	12.21 ± 0.19 a	4.34 ± 0.09 b	5.77 ± 0.38 d	14.92 ± 0.06 a	4104.90 ± 16.31 a
*Eyprepocnemis* *smaragdipes*	8.95 ± 0.28 d	58.09 ± 0.09 b	8.88 ± 0.11 b	4.46 ± 0.04 b	7.14 ± 0.16 c	12.48 ± 0.32 b	3611.77 ± 11.80 b
*Acrida madecassa*	7.86 ± 0.12 c	57.15 ± 0.52 c	13.34 ± 1.59a	8.37 ± 0.08 a	8.37 ± 0.56 b	4.91 ± 0.02 c	3627.17 ± 164.65 b
*p*-value	0.0001	0.0001	0.0001	0.0001	0.0001	0.0001	0.0001
F-value	874.20	362.90	17.16	2379.30	74.17	327.20	28.3
df	3, 8	3, 8	3, 8	3, 8	3, 8	3, 8	3, 8
RDI (g/100g DM) by USDA for							
Children 5 to 10 years	NR	24	70	4	15	220	1800
Adult males	NR	46	95	6	24	230	2550
Adult females	NR	56	70	6	24	230	200

Results are expressed as mean values ± standard deviation of three determinations on a dry matter (DM) basis. Means with different letters in each column significantly differ at *p* < 0.05. RDI means recommended daily intake, USDA refers to the United States Department of Agriculture [40], and NR means not reported.

**Table 4:** Comparison of mineral compositions of *Paracinema tricolor*, *Oxya hyla*, *Eyprepocnemis smaragdipes,* and *Acrida madecassa* and the conventional protein sources typically consumed by Malagasy people on a dry matter basis (mean ± SE) and recommended daily intake.

Grasshopper Species	Mineral Elements (mg/100 g)								
	Magnesium (Mg)	Iron (Fe)	Calcium (Ca)	Copper (Cu)	Phosphorus (P)	Zinc (Zn)	Potassium (K)	Manganese (Mn)	Sodium (Na)
*Paracinema tricolor*	123.40 ± 3.34 a	20.39 ± 0.19 a	366.17 ± 15.67 a	2.62 ± 0.01 b	859.77 ± 14.71 a	29.80 ± 0.81 a	1210.62 ± 17.37 a	12.57 ± 0.06 a	264.70 ± 5.77 a
*Oxya hyla*	103.56 ± 3.88 b	7.67 ± 0.65 c	148.12 ± 10.10 b	5.80 ± 0.44 a	314.68 ± 7.39 c	16.75 ± 0.25 c	1014.31 ± 22.49 b	3.53 ± 0.15 c	151.95 ± 6.11 b
*Eyprepocnemis smaragdipes*	105.72 ± 7.63 b	19.07 ± 0.37 b	36.78 ± 4.09 d	1.82 ± 0.06 c	765.51 ± 24.52 b	23.75 ± 3.02 b	844.38 ± 23.56 c	7.22 ± 0.41 b	40.75 ± 1.15 c
*Acrida madecassa*	74.21 ± 4.14 c	4.95 ± 0.34 d	65.21 ± 4.20 c	1.79 ± 0.08 c	216.28 ± 9.08	14.85 ± 0.79 c	825.56 ± 26.14 c	0.81 ± 0.05 d	82.93 ± 4.16 c
F	49.10	989.90	698.40	211.50	1247.00	53.69	188.50	1544.00	25.96
*p*	<0.0001	<0.0001	<0.0001	<0.0001	<0.0001	<0.0001	<0.0001	<0.0001	=0.0002
df	3, 8	3, 8	3, 8	3, 8	3, 8	3, 8	3, 8	3, 8	3, 8
Mutton	24.00	2.00	22.00	0.13	232.00	4.63	336.00	0.02	403.00
Turkey	30.00	1.10	14.00	0.09	263.00	2.43	239.00	0.03	103.00
Mud crab	47.65	1.21	24.61	0.91	296.00	3.80	29.50	0.03	26.85
Tilapia fish	34.00	0.69	16.70	0.08	392.00	0.41	380.00	0.81	56.00
Pork	25.9	1.40	37.90	0.10	323.00	3.20	504.30	0.01	83.70
Eggs	50.30	7.30	54.00	0.30	122.00	5.40	578.60	0.01	595.40
Chicken	58.80	2.60	32.20	1.40	172.00	3.90	555.70	0.11	205.80
Beef	39.80	4.30	18.70	0.20	263.00	8.40	624.70	0.02	138.00
RDI (mg/100 g DM) by the USDA for Children									
0–3 years	20–50	7	700	0.4–1	100–460	2–3	2000	0.003–1.2	1200
4–8 years	65	10	1000	1–1.5	500	4	2300	1.5	1500
9–13 years	100–135	8–10	1000–1300	1–2	1250	4–6	2500	1.66	1800
Adults	320–429	10–18	1000–1200	1.5–3.0	700	8–11	2300–3400	1.8–2.3	1500

The results are expressed as mean values ± standard deviation of three determinations on a dry matter (DM) basis. Means with different letters in each column significantly differ at *p* < 0.05. The mineral element data for conventional sources were obtained from the USDA (United States Department of Agriculture) [40]. RDI means recommended daily intake and USDA refers to the United States Department of Agriculture [40].

**Table 5:** Fatty acid composition of *Paracinema tricolor*, *Oxya hyla*, *Eyprepocnemis smaragdipes*, and *Acrida madecassa* on a dry matter basis (mean ± SE).

Fatty Acid	*Paracinema tricolor*	*Oxya hyla*	*Eyprepocnemis smaragdipes*	*Acrida madecassa*	*p*-Value	F-Value	df
Lauric acid (C12:0)	0.06± 0.01 c	0.42 ± 0.05 a	0.30 ± 0.01 b	0.03 ± 0.01 c	163.90	0.0001	3, 8
Myristic acid (C14:0)	1.10 ± 0.25 b	3.32 ± 0.43 a	0.56 ± 0.02 c	0.42 ± 0.04 c	87.71	0.0001	3, 8
Pentadecanoic acid (C15:0)	0.26 ± 0.07 b	0.55 ± 0.11 a	0.15± 0.01 b	0.29 ± 0.03 b	18.25	0.0006	3, 8
Palmitic acid (C16:0)	11.59 ± 0.93 c	13.74 ± 0.55 b	10.55 ± 0.55 c	17.26 ± 0.45 a	63.11	0.0001	3, 8
Margaric acid (C17:0)	1.38 ± 0.36 a	1.14 ± 0.08 a	0.42 ± 0.03 b	0.41 ± 0.06 b	21.35	0.0004	3, 8
Stearic acid (C18:0)	10.25 ± 0.04 b	11.30 ± 0.86 a	10.10 ± 0.40 b	8.09 ± 0.20 c	23.18	0.0003	3, 8
Arachidic acid (C20:0)	1.57 ± 0.26 b	1.54 ± 0.15 b	1.30 ± 0.06 b	2.28 ± 0.06 a	21.95	0.0003	3, 8
Behenic acid (C22:0)	1.06 ± 0.07 a	0.52 ± 0.01 b	0.40 ± 0.02 b	1.00 ± 0.02 a	24.14	0.0001	3, 8
Lignoceric acid (C24:0)	0.04 ± 0.01 c	0.85 ± 0.01 b	1.31 ± 0.01 a	0.02 ± 0.01 d	12,100.00	0.0001	3, 8
Alpha-linolenic acid (C 18:3(n-3))	0.50 ± 0.10 a	0.40 ± 0.10 a	0.34 ± 0.01 a	0.32 ± 0.01 a	3.88	0.0555	3, 8
Linoleic acid (C18:2(n-6))	43.73 ± 0.70 a	39.06 ± 0.38 b	38.92 ± 0.66 b	39.65 ± 0.61 b	43.20	0.0001	3, 8
Oleic acid (C18:1(n-9))	28.46 ± 0.40 c	27.16 ± 0.64 d	35.65 ± 0.55 a	30.23 ± 0.34 b	168.70	0.0001	3, 8
Monounsaturated fatty acids (MUFAs)	28.46 ± 0.40 c	27.16 ± 0.64 d	35.65 ± 0.55 a	30.23 ± 0.34 b	168.70	0.0001	3, 8
Polyunsaturated fatty acids (PUFAs)	44.23 ± 0.04 a	39.46 ± 0.37 b	39.26 ± 0.62 b	39.97 ± 0.64 b	46.06	0.0001	3, 8
Saturated fatty acids (SFAs)	27.31 ± 0.34 c	33.38 ± 0.45 a	25.09 ± 0.71 d	29.80 ± 0.52 b	57.86	0.0001	3, 8
Essential fatty acids (EFAs)	44.23 ± 0.04 a	39.46 ± 0.37 b	39.26 ± 0.62 b	39.97 ± 0.64 b	46.06	0.0001	3, 8
PUFA/SFA	1.62	1.18	1.56	1.34			

The results are expressed as mean values ± standard deviation of three determinations on a dry matter (DM) basis. Means with different letters in each row significantly differ at *p* < 0.05.

**Table 6:** Amino acid profile (µg/100 mg) of *Paracinema tricolor, Oxya hyla*, *Eyprepocnemis smaragdipes*, and *Acrida madecassa*.

Amino Acid	*Paracinema tricolor*	*Oxya hyla*	*Eyprepocnemis smaragdipes*	*Acrida madecassa*	*p*-Value	F-Value	df	RDA by USDA for			
Essential amino acids								Infants (3–4 months)	Children (~2 years)	Children (10–12 years)	Adults
Histidine	14.60 ± 0.20 a	5.04 ± 0.08 b	4.79 ± 0.10 b	4.75 ± 0.01 b	0.0001	581.70	3, 8	1.6	1.9	1.9	1.1
Isoleucine	59.28 ± 0.04 a	45.24 ± 0.25 b	43.74 ± 0.09 c	43.11 ± 0.10 d	0.0001	9037.00	3, 8	4.0	2.8	2.8	1.3
Lysine	24.83 ± 0.31 a	16.06 ± 0.10 b	15.58 ± 0.13 c	15.31 ± 0.17 c	0.0001	1650.00	3, 8	6.0	5.8	4.4	1.6
Methionine	23.02 ± 0.03 a	21.39 ± 0.04 b	20.67 ± 0.06 b	20.91 ± 0.77 b	0.0002	22.70	3, 8	NR	NR	NR	NR
Valine	39.81 ± 0.69 c	42.82 ± 0.08 a	41.30 ± 0.10 b	40.67 ± 0.06 b	0.0001	43.05	3, 8	5.4	3.5	2.5	1.3
Phenylalanine	34.75 ± 0.22 a	33.80 ± 0.27 b	32.65 ± 0.19 c	32.15 ± 0.12 d	0.0001	96.13	3, 8	NR	NR	NR	NR
Leucine	98.45 ± 0.23 a	81.94 ± 0.59 b	78.70 ± 0.24 c	77.17 ± 0.15 d	0.0001	2375.00	3, 8	9.3	6.6	4.4	1.9
Nonessential amino acid											
Arginine	18.60 ± 0.07 a	14.36 ± 0.31 b	14.02 ± 0.16 bc	13.75 ± 0.05 c	0.0001	481.20	3, 8	NR	NR	NR	NR
Glutamine	2.81 ± 0.04 a	1.83 ± 0.03 b	1.75 ± 0.02 c	1.71 ± 0.02 c	0.0001	1391.00	3, 8	NR	NR	NR	NR
Glutamic acid	13.03 ± 0.07 a	11.72 ± 0.07 b	11.31 ± 0.06 c	11.14 ± 0.07 d	0.0001	559.50	3, 8	NR	NR	NR	NR
Proline	26.29 ± 0.14 d	31.54 ± 0.06 a	30.44 ± 0.07 b	30.03 ± 0.10 c	0.0001	1605.00	3, 8	NR	NR	NR	NR
Tyrosine	32.49 ± 0.13 a	25.26 ± 0.35 b	24.49 ± 0.05 c	24.09 ± 0.10 d	0.0001	1266.00	3, 8	NR	NR	NR	NR
Hydroxyproline	12.05 ± 0.20 a	9.83 ± 0.42 b	9.33 ± 0.14 c	9.22 ± 0.03 c	0.0001	90.9 3	3, 8	NR	NR	NR	NR

The results are expressed as mean values ± standard deviation of three determinations on a dry matter (DM) basis. Means with different letters in each row significantly differ at *p* < 0.05. RDI means recommended daily intake and USDA refers to the United States Department of Agriculture [40]. NR means not reported.

**Table 7:** Vitamin content of *Paracinema tricolor*, *Oxya hyla*, *Eyprepocnemis smaragdipes*, and *Acrida madecassa* and recommended daily intake.

Grasshopper Species	Vitamin A (μg/100)	Vitamin E (IU/kg)	Vitamin C (Ascorbic Acid) (mg/100 g)	Vitamin B1 (Thiamine) (mg/100 g)	Vitamin B2 (Riboflavin) (mg/100 g)	Vitamin B3 (Niacin) (mg/100 g)	Vitamin B9 (Folic Acid) (mg/100 g)
*Paracinema tricolor*	334.21 ± 0.06 a	47.61 ± 0.06 a	0.16 ± 0.02 a	0.83 ± 0.06 a	1.50 ± 0.64 a	3.34 ± 0.06 a	1.49 ± 0.10 a
*Oxya hyla*	293.42 ± 0.31 b	41.79 ± 0.04 b	0.13 ± 0.02 ab	0.70 ± 0.03 b	1.64 ± 0.06 a	2.92 ± 0.07 b	1.29 ± 0.06 b
*Eyprepocnemis smaragdipes*	283.09 ± 0.12 c	40.39 ± 0.06 c	0.11 ± 0.02 b	0.66 ± 0.02 b	1.60 ± 0.05 a	2.79 ± 0.04 c	1.16 ± 0.05 bc
*Acrida madecassa*	278.70 ± 0.42 d	39.70 ± 0.07 d	0.11 ± 0.02 b	0.65 ± 0.03 b	1.58 ± 0.05 a	1.75 ± 0.03 d	1.07 ± 0.03 c
F	2679.00	875.30	4.63	16.61	0.102	486.30	21.60
*p*	<0.0001	<0.0001	=0.0369	=0.0009	=0.9570	<0.0001	=0.0003
df	3, 8	3, 8	3, 8	3, 8	3, 8	3, 8	3, 8
Recommended daily intake (RDI)	500.00–700.00 μg	7.50–10.00 mg	45.00 mg	1.20–1.30 mg	1.10–1.30 mg	14.00–16.00 mg	0.40 mg

The results are expressed as mean values ± standard deviation of three determinations on a dry matter (DM) basis. Means with different letters in each column significantly differ at *p* < 0.05. RDI means recommended daily intake, and USDA refers to the United States Department of Agriculture [40].

## Data Availability

The original contributions presented in this study are included in the article; further inquiries can be directed to the corresponding author.
